# A multi-center clinical investigation on invasive *Streptococcus pyogenes* infection in China, 2010–2017

**DOI:** 10.1186/s12887-019-1536-1

**Published:** 2019-06-05

**Authors:** Chun-Zhen Hua, Hui Yu, Hong-Mei Xu, Lin-Hai Yang, Ai-Wei Lin, Qin Lyu, Hong-Ping Lu, Zhi-Wei Xu, Wei Gao, Xue-jun Chen, Chuan-Qing Wang, Chun-mei Jing

**Affiliations:** 1 Division of Infectious Diseases, The Children’s Hospital, Zhejiang University School of Medicine, Hangzhou, 310003 People’s Republic of China; 2 Division of Infectious Diseases, Children’s Hospital of Fudan University, Shanghai, 201102 People’s Republic of China; 3 Division of Infectious Diseases, Chongqing Medical University Affiliated Children’s Hospital, Chongqing, 400014 People’s Republic of China; 4 Department of Cardiology, Shanxi Children’s Hospital, Taiyuan, 030013 People’s Republic of China; 5 Division of Infectious Diseases, Qilu Children’s Hospital of Shandong University, Jinan, 250022 People’s Republic of China; 6 The Intensive Care Unit, Ningbo Women and Children’s Hospital, Ningbo, 315012 People’s Republic of China; 7 The intensive Care Unit, Taizhou Hospital of Zhejiang Province, Linhai, 317000 People’s Republic of China; 8 Division of Infectious Diseases, The Second Affiliated Hospital &Yuying Children’s Hospital of Wenzhou Medicial University, Wenzhou, 325027 People’s Republic of China; 9 Division of Infectious Diseases, Kaifeng Children’s Hospital, Kaifeng, 475000 People’s Republic of China; 10 Department of Clinical Laboratory, The Children’s Hospital, Zhejiang University School of Medicine, Hangzhou, 310003 People’s Republic of China; 11 Department of Clinical Laboratory, Children’s Hospital of Fudan University, Shanghai, 201102 People’s Republic of China; 12 Department of Clinical Laboratory, Chongqing Medical University Affiliated Children’s Hospital, Chongqing, 400014 People’s Republic of China

**Keywords:** *Streptococcus pyogenes*, invasive infection, Group a streptococcus, Children, Streptococcal toxin shock syndrome

## Abstract

**Background:**

Invasive *S. pyogenes* diseases are uncommon, serious infections with high case fatality rates (CFR). There are few publications on this subject in the field of pediatrics. This study aimed at characterizing clinical and laboratory aspects of this disease in Chinese children.

**Patients and methods:**

A retrospective study was conducted and pediatric in-patients with *S. pyogenes* infection identified by cultures from normally sterile sites were included, who were diagnosed and treated in 9 tertiary hospitals during 2010–2017.

**Results:**

A total of 66 cases were identified, in which 37 (56.1%) were male. The median age of these patients, including 11 neonates, was 3.0 y. Fifty-nine (89.4%) isolates were determined from blood. Fever was the major symptom (60/66, 90.9%) and sepsis was the most frequent presentation (64/66, 97.0%, including 42.4% with skin or soft tissue infections and 25.8% with pneumonia. The mean duration of the chief complaint was (3.8 ± 3.2) d. Only 18 (27.3%) patients had been given antibiotics prior to the hospitalization. Among all patients, 15 (22.7%) developed streptococcal toxin shock syndrome (STSS). No *S. pyogenes* strain was resistant to penicillin, ceftriaxone, or vancomycin, while 88.9% (56/63) and 81.4% (48/59) of the tested isolates were resistant to clindamycin and erythromycin respectively. Most of the patients were treated with β-lactams antibiotics and 36.4% had been treated with meropenem or imipenem. Thirteen (19.7%) cases died from infection, in which 9 (13.6%) had complication with STSS.

**Conclusions:**

Invasive *S. pyogenes* infections often developed from skin or soft tissue infection and STSS was the main cause of death in Chinese children. Ongoing surveillance is required to gain a greater understanding of this disease.

## Background

Group A streptococcus (GAS), such as *Streptococcus pyogenes* (*S. pyogenes*), is a major human pathogen and causes a wide range of diseases, from mild skin and soft tissue infections, pharyngitis, tonsillitis to severe invasive diseases in humans [1]. A case of invasive GAS (iGAS) disease is defined as GAS is isolated from a normally sterile body site or from a wound specimen obtained from a patient with necrotizing fasciitis or streptococcal toxic shock syndrome (STSS) [2]. Epidemiology of iGAS infection is highly variable in different population and the annual incidences varied from 2.5/100,000 to 101/100,000 in children around the world [2–5]. The iGAS disease causes serve infection associated with high mortality, especially in those who developed STSS [1–5]. Nonetheless, no active surveillance on iGAS infections was conducted and data regarding clinical characteristics and risk factors of such pediatric patients are limited in China. Though *S. pyogenes*, the most common member of GAS, was rarely isolated from normally sterile site, it was an important pathogen due to causing life-threaten infection which were associated with hospital-patient disputes in China. The aim of this work is to improve the understanding of the clinical and laboratory characteristics and treatment of invasive *S. pyogenes* disease (ISPD) in children at a regional level.

## Methods

### Setting and patients

Retrospectively, patients with at least one positive culture from normally sterile body sites for *S. pyogenes* were included at 9 tertiary hospitals in 9 cities in China. Every institution had 300–1500 pediatric beds and approximately 10,000–39,000 hospitalizations each year. The patients who were admitted during the years 2010–2017 and with a disease onset at ≤18 years of age were included in the study. Patients’ demographics, type of infection, clinical presentation, treatment, microbiological data, hospital stay and evolution were collected from electronic medical records.

#### Identification of organism and drug-susceptibility

Each organism was identified using the Vitek system GPI card (Mérieux, France) $\mathrm{or}$Maldi-TOF mass spectrometry analysis (Bruck, Germany). Antimicrobial susceptibility test was performed using Kirby-Bauer method according to the criteria of the Clinical and Laboratory Standards Institute at the 9 institutions. Repeated *S. pyogenes* isolation from different samples of the same patient was considered as one *S. pyogenes* clone.

#### Statistical analysis

The demography of the patients was summarized by mean, standard deviation (SD), interquartile range and proportion as appropriate.

#### Ethics

This study was performed under the institutions opt-out passive consent policy and approved by the ethics committee and the Institutional Board of Privacy and Security at the hospitals (2018-IEC-047). Patients and guardians wishing to withdraw from the study were able to contact the principal investigator through information provided on notifications publically posted by the institution’s ethics committee.

## Results

### Patients’ baseline characteristics

A total of 66 hospitalized patients with culture-confirmed ISPD were included and 37 (56.31%) were male. Age of the patients ranged from 2 h to 15 y and the median age was 3.0 y (p25–75: 0.48–8 y). Eleven (16.7%) were neonate. Thirteen (19.7%) had underlying condition. Forty-eight (72.7%) had not been prescribed with any antibiotics before specimen were collected for culture. Patients’ underlying condition and prior antibiotics use were summarized in Table 1.

**Table 1:** The underlying condition and prior antibiotics use in the 66 patients with invasive *S. pyogenes* infection

Underlying condition	N	%	Previous antibiotics	N	%
Leukemia	2	3.0	Azithromycin, IV × 2–5 d	7	10.6
Varicella	2	3.0	Cephalosporins, Oral × 3–5 d	3	4.6
Skin burn	2	3.0	Azithromycin, Oral ×3 d	1	1.5
Skin hemangioma	2	3.0	Erythromycin, Oral ×3 d	1	1.5
Nephrotic syndrome	1	1.5	Piperacillin-tazobactam, IV × 1 d	1	1.5
Still’s disease	1	1.5	Erythromycin, IV × 1 d;	1	1.5
			Clindamycin, IV × 1 d		
Cerebral trauma	1	1.5	Azithromycin, IV × 5 d;	1	1.5
			Clindamycin, IV × 1 d		
Craniocerebral trauma	1	1.5	Meropenem + vancomycin +	1	1.5
			Metronidazole, IV × 3 d		
Cerebrospinal fluid rhinorrhea	1	1.5	Meropenem, IV × 3 d;	1	1.5
			Lynezoliane, IV × 1 d		
None	53	80.5	Piperacillin-tazobactam, IV × 1 d;	1	1.5
			Clindamycin, IV × 4 d;	1	1.5
			Vancomycin + Mezlocillin, IV × 1d		
			None	47	71.3
Total	66	100	Total	66	100

Note: IV, intravenous

#### Clinical characteristics

Of all 66 patients, disease course was 3.8 ± 3.2 d (2 h − 20 d). Sixty (90.9%) patients had fever with mean temperature as 39.4° ± 0.9 °C. Of the other six patients who did not have fever, 4 (6.1%) were neonates and 2 (3.0%) were infants at 1 month of age. Most frequent clinical manifestation was sepsis (64 cases, 97.0%), 59 of which were blood culture confirmed. Fifteen (22.7%) developed STSS. One patient had been misdiagnosed as Kawasaki disease because of rash, conjunctival congestion, strawberry tongue, cervical lymphadenopathy and peeling around the anus. Lumbar puncture was performed in 23 (34.8%) patients and 8 (12.1%) were identified as meningitis by laboratory examination of cerebrospinal fluid, though only 2 of the 8 had a positive result as *S. pyogenes* by culture. One patient had concomitant infection with both *S. pyogenes* and *Streptococcus pneumonia* in blood stream. Nineteen (28.8%) patients had multi-organ failure. The clinical manifestation was shown in Table 2.

##### Laboratory results

(1) Blood Routine Initial leukocyte count was quite variable. Leukopenia was presented in 7 (10.6%) patients and leukocytosis was presented in 44 (66.7%) patients. Fifty-nine (89.4%) patients had CRP levels higher than normal value (10 mg/L). Procalcitonin levels in sera were detected in 40 patients and 33 (82.5%) had levels higher than normal value (0.5 mg/L). The blood routine, C reaction protein levels, and procalcitonin levels in the 66 patients were shown in Table 3. (2) Isolates and antibiotic-resistance Sixty-six strains of *S. pyogenes* were identified in 66 patients. The vast majority of isolates, 59 (89.4%), were obtained from blood, with the remaining from cerebrospinal fluid (2, 3.0%), joints (2, 3.0%), pleural fluid (2, 3.0%) and peritoneal fluid (1, 1.5%). *S. pyogenes* was isolated from multiple sites for some patients, such as 3 (4.5%) patients from both blood and pyogenic fluids from skin and soft tissue, two (3.0%) patients from both blood and purulent exudation from tonsil, and 2 (3.0%) from both blood and articular fluid. All tested strains were phenotypically sensitive to penicillin, ceftriaxone, cefotaxime, vancomycin, and lynezolid. The resistant rate to erythromycin, tetracycline, clindamycin and trimethoprim-sulfamethoxazole were 88.9, 81.5, 81.4 and 72.0% respectively. Table 4 showed the resistant rates of *S. pyogenes* strains against 10 antibiotics during 2010–2017 in the 9 tertiary hospitals.

##### Treatment and outcome:

The mean duration of treatment in these 66 patients was 16.3 ± 13.2 d (range: 1–60 days). The medium hospital stay was 17 d (p25–75: 6–23 days). Of the 66 patients, 25 (37.9%) were admitted to pediatric intensive care unit (PICU) and the median ICU stay was 5 days. The details of the therapy in 66 patients were summarized in Table 2. Two (3.0%) patients did not receive any antibiotics because of abandoning therapy or death within 2 h of admission. Fourteen (21.2%) were treat with single antibiotic, and 50 (75.8%) had been treated with 2 or more than 2 antibiotics, either in combination or sequentially. Eighteen (27.3%) cases required surgical debridement and 18 (27.3%) cases received intravenous immunoglobulin. Seventeen (25.8%) patients received mechanical ventilation for a median duration of 3 days. Fifty-three (80.3%) patients survived and 7 (13.2%) of which had sequelae including 4 (7.5%) with limp, 2 (3.8%) with hemiplegia and 1 (1.9%) with epilepsy. The total fatalities were 13 (19.7%) cases, within which 7 (53.8%) died in 24 h of admission and 10 (76.9%) died in 72 h of admission. The case fatality ratio (CFR) was 53.3% in the case of STSS. Only 1 (1.5%) patient who died had an underlying condition as cerebral palsy and was abandoned. Secondary transmission was not found in this study. Hospital-patient disputes occurred in 6 (9.1%) patients.

**Table 2:** The clinical manifestation and antibiotics for therapy in the 66 patients with invasive *S. pyogenes* infection

Clinical manifestation	N	%	Treatment with antibiotics	N	%
Meningitis with cerebral trauma	1	1.5	^a^ Penicillins or/ and Cephalosporins	27	40.9
Pneumonia with pleural effusion	1	1.5	Carbapenems + vancomycin	7	10.6
Sepsis (64 cases, 97.0%)			Carbapenems, penicillin	3	4.6
Skin / soft tissue infections	21	31.8	Vancomycin + cephalosporins, Cephalosporins	3	4.6
Severe pneumonia (4 with pleural effusion)	10	15.2	Vancomycin	2	3.0
Suppurative arthritis and osteomyelitis	6	9.1	Carbapenems	2	3.0
Both Pneumonia and skin / soft tissue infections (1 with pleural effusion)	6	9.1	Carbapenems, Cephalosporins	2	3.0
Suppurative tonsillitis	3	4.5	Vancomycin + cephalosporins	2	3.0
Meningitis	3	4.5	Carbapenems + vancomycin, vancomycin	2	3.0
Skin / soft tissue infections, suppurative arthritis, osteomyelitis and necrotising fasciitis	2	3.0	Carbapenems + vancomycin, Vancomycin + cephalosporins	2	3.0
Both pneumonia and meningitis	1	1.5	Vancomycin + cephalosporins, Penicillin	2	3.0
Both skin / soft tissue infections and meningitis	1	1.5	^b^Other regimen	10	15.3
Both mastoiditis and otitis media	1	1.5	No antibiotics	2	3.0
Without identified focus	10	15.3			
Total	66	100	Total	66	100

^a^Penicillins (4 cases); Cephalosporins (13 cases); Penicillins + Cephalosporins (5 cases); Cephalosporins, penicillins (5 cases)

^b^Clindamycin, azithromycin (1 case); azithromycin, vancomycin (1 case); Carbapenems+ linezolid, penicillin + linezolid (1 case); Carbapenems + vancomycin, lynezolid (1 case); Carbapenems + vancomycin, penicillin + lynezolid (1 case); Carbapenems + vancomycin, cephalosporins (1 case); Carbapenems, lynezolid (1 case); Carbapenems + vancomycin, vancomycin, cephalosporins (1 case); Vancomycin + cephalosporins, penicillin (1 case); Vancomycin + penicillin, penicillin (1 case)

**Table 3:** The Leukocyte count, Neutrophils count, C reaction protein levels, and procalcitonin levels in the 66 patients with invasive *S. pyogenes* infection

	Min	Mean ± SD/ median (P_25_-P_75_)	Max
Leukocyte count	1.1 × 10^9^/L	(17.6 ± 11.8) × 10^9^/L	50.4 × 10^9^/L
Neutrophils count	0.2 × 10^9^/L	(14.0 ± 10.8) × 10^9^/L	41.5 × 10^9^/L
SCRP	2.6	103 (60–142) mg/L	> 180 mg/L
Procalcitonin^a^	0.1	60 (1.9–73.0) mg/L	> 200 mg/L

^a^Procalcitonin levels were test in 40 patients

**Table 4:** Resistance of *S. pyogenes* strains to 10 antibiotics in 2011–2017^a^

Antibiotics (N)	S (N, %)	I (N,%)	R (N,%)
Penicillin G (63)	63 (100.0)	0 (0.0)	0 (0.0)
Ceftriaxone (36)	36 (100.0)	0 (0.0)	0 (0.0)
Cefotaxime (54)	54 (100.0)	0 (0.0)	0 (0.0)
Vancomycin (63)	63 (100.0)	0 (0.0)	0 (0.0)
Lynezolid (51)	51 (100.0)	0 (0.0)	0 (0.0)
Erythromycin (63)	6 (9.5)	1 (1.6)	56 (88.9)
Clindamycin (59)	10 (16.9)	1 (1.7)	48 (81.4)
Levofloxacin (58)	55 (94.8)	2 (3.5)	1 (1.7)
SXT (25)	6 (24.0)	1 (4.0)	18 (72.0)
Tetracycline (38)	2 (5.3)	5 (13.2)	31 (81.5)

*S* sensitive, *I* intermediate, *R* resistant, *SXT* sulfamethoxazole-trimethoprim

^a^Drug-susceptibility test was not performed in three strains owing to the death of the three patients. Antibiotics tested in the nine hospitals differed during these years

## Discussion

ISPDs are uncommon but serious infections with high CFR. There are few publications on this subject at a regional level, particularly in the field of pediatrics. In the current study, only 66 pediatric patients were identified based on culture from 9 tertiary hospitals in 8 years. The low case number could be due to prior antibiotics usage that interfered with the etiological diagnosis of this disease. Because most of the patients were previously healthy children and the CFR was relative higher, hospital-patient disputes occurred in 9.1% of all patients, which caused huge trouble for the hospital. Thus, early recognition of this disease for effective treatment is really important. ISPDs have a broad and evolving clinical spectrum. Previous reports identified skin as a potential source for main trigger that leads to clinical manifestation of bacteremia or sepsis [6, 7], which were in accordance with our findings that 31.8% of the patients had skin or soft tissue infections as predisposing factors, indicating that pediatricians and emergency physicians must be aware of this possibility when treat skin or soft tissue infection. Zachariadou et al. [8] reported that varicella and streptococcal pharyngotonsillitis was the main predisposing factors in children, which was not supported in the present study. Neonates and infants < 1 y are susceptible to *S. pyogenes* infections [5], and our study showed that 16.7% of all patients were neonates, which may be associated with their mother being a carrier or infected with this bacteria [9]. Most of the patients had a maximum temperature > 39.0 °C and the fever tended to be ardent. High levels of leukocyte counts, CRP and procalcitonin concentration were found in most patients, which suggests that *S. pyogenes* infection is associated with relatively serious inflammation.

Determination of resistance to antibiotics in strains helps antibiotic choice in therapy. Even at present, penicillin-nonsusceptible *S. pyogenes* are absence or extremely rare. Thus, penicillin remains the first-choice for treatment and is also a surrogate for ampicillin, amoxicillin, cefotaxime and ceftriaxone, in treating infectious disease caused by this bacterium. In the present study, all of the isolates were sensitive to beta-lactams. Most of the patients were cured with beta-lactams in therapy, which indicates the consistency of the antimicrobial activity of beta-lactams in vitro and in vivo. Macrolides are important alternatives for allergic patients and lincosamides are recommended together with beta-lactams in treating invasive infections [10, 11]. For reasons that are not completely clear, macrolides-resistance is highly variable across countries. In Norway during 2010–2014, < 4% of the included *S. pyogenes* were resistant to erythromycin [12]. Chochua et al. reported that of the 1454 invasive isolates, 12.7% were nonsusceptible to erythromycin [13]. During 2008–2013 in Finland, an increase of erythromycin resistance (1.9 to 8.7%) and clindamycin (0.9 to 9.2%) were found [14]. Wajima et al. reported that 54.4% of the 283 invasive isolated were erythromycin-resistant [15]. In Asia, macrolides-resistance even higher. Lu et al. reported that 93.5 and 94.2% of the strains isolated from 2009 to 2016 were resistant to erythromycin and clindamycin, respectively [16]. Similarly, we found that 88.9 and 81.4% of the tested strains were resistant to erythromycin and clindamycin, which indicated that macrolides would not be the alternatives even for penicillin-allergic patients with *S. pyogenes* infection in China. The high macrolides-resistance of *S. pyogenes* isolates may associate with wide usage of macrolides in this population. Addition of a high dosage of clindamycin is recommended because of its excellent tissue penetration and improving the outcome by modulating virulence factors of clindamycin-susceptible and clindamycin-resistant *S. pyogenes* [11], In addition, clindamycin is an important adjunctive antibacterial, however, in the current study, most of the patients did not receive clindamycin, the possible explanation is that pediatrician choose antibiotics for therapy were based on antibiotic-resistance only and did not realize it’s role on inhibiting virulence factors of the bacteria.

The onset and progression of ISPD can be rapid, and the associated mortality is high especially those complicated with STSS [17]. Given the rapid clinical progression, effective management of ISPD hinges on early recognition of the disease and prompt initiation of supportive care together with antibacterial therapy and early surgical debridement of infected tissue. Early institution of intravenous immunoglobulin therapy should be considered in cases of STSS and severe invasive infection, including necrotizing fasciitis [9, 11]. However, only 27.3% of the patients received intravenous immunoglobulin, which may associate with the mortality in the present study was higher than in other study which were not more than 15% [2–4, 7, 18]. In cases of severe invasive infections, it is often difficult to distinguish among bacterial infections before cultures become available and so antibiotics choice must include coverage of both of Gram-positive and Gram-negative bacteria. Rapid antigen detection tests [19] and PCR assay will help antibiotic prescriptions in the management of life-threatening *S. pyogenes* infections. Linder et al. [20] reported that compared with non-immunocompromised patients, immunocompromised patients are more likely to develop STSS and have a higher mortality, which were not identified in the present study.

There are limitations in this study. This study is retrospective based on database in China. The number of included cases with ISPD was small, thus, the results should be interpreted carefully. Usually, specific emm-genotypes are association with ISPD, for example, emm 1 are the leading cause of invasive disease worldwide [8, 12, 19, 21, 22], However, genotypes of *S. pyogenes* were not performed in the study. Hereafter, a nationwide survey is required to clarify the epidemiology, risk factors, clinical and microbiological characteristics of *S. pyogenes* invasion disease in children in China. Ongoing surveillance is required in order to undertake appropriate control measures and gain a greater understanding of this disease.

## Conclusions

Invasive *S. pyogenes* infections often developed from skin or soft tissue infection, fever was the major symptom, sepsis was the most frequent presentation and STSS was the main cause of death in Chinese children. Ongoing surveillance is required to gain a greater understanding of this disease in China.

## Abbreviations

CFR: Case fatality rate
CRP: C-reactive protein
GAS: Group A streptococcus
iGAS: Invasive GAS infection
PCR: Polymerase chain reaction
PICU: Pediatric intensive care unit
*S. pyogenes*: *Streptococcus pyogenes*
SD: Standard deviation
STSS: Streptococcal toxic shock syndrome
SXT: Sulfamethoxazole-trimethoprim
